# Supplementary material for: Assessment of pathogens in flood waters in coastal rural regions: Case study after Hurricane Michael and Florence
Source: PLoS One. 2023 Aug 4;18(8):e0273757. doi: 10.1371/journal.pone.0273757 (PMC10403080; doi:10.1371/journal.pone.0273757)
Supplement: S1 Table — (DOCX) [file pone.0273757.s001.docx]

**Assessment of pathogens in flood waters in coastal rural regions: Case study after Hurricane Michael and Florence**

Moiz Usmani^1^, Sital Uprety^2^, Nathan Bonham^1^, Yusuf Jamal^1^, Yuqing Mao^2^, Daisuke Sano^3^, Joanna Shisler^4,5^, Avinash Unnikrishnan^6^, Thanh H. Nguyen^2^, Antarpreet Jutla^1^*

^1^Environmental Engineering Sciences, University of Florida, Gainesville, FL, USA

^2^Department of Civil and Environmental Engineering, University of Illinois at Urbana-Champaign, Urbana, IL, USA

^3^Department of Civil and Environmental Engineering, Tohoku University, Sendai, Japan

^4^Institute for Genomic Biology, University of Illinois at Urbana-Champaign, Urbana, IL, USA

^5^Department of Microbiology, University of Illinois at Urbana-Champaign, Urbana, IL, USA

^6^Civil and Environmental Engineering, Portland State University, Portland, OR, USA

*Corresponding author: ajutla@ufl.edu

Supplementary Material Table 1

Table S1: The number of filters used for each sample.

| Sampling site | Replicate | # of filters of this replicate | Total # of filters at this site |
| --- | --- | --- | --- |
| U1 | A | 4 | 12 |
|  | B | 4 |  |
|  | C | 4 |  |
| U2 | A | 2 | 6 |
|  | B | 2 |  |
|  | C | 2 |  |
| U3 | A | 2 | 5 |
|  | B | 1 |  |
|  | C | 2 |  |
| U5 | A | 3 | 8 |
|  | B | 5 |  |
| U6 | A | 4 | 8 |
|  | B | 4 |  |
| U7 | A | 4 | 8 |
|  | B | 4 |  |
| U8 | A | 3 | 9 |
|  | B | 3 |  |
|  | C | 3 |  |
| U10 | A | 2 | 7 |
|  | B | 2 |  |
|  | C | 3 |  |
| F1 | A | 5 | 13 |
|  | C | 4 |  |
|  | D | 4 |  |
| F2 | A | 4 | 12 |
|  | B | 3 |  |
|  | C | 5 |  |
| F3 | A | 4 | 10 |
|  | B | 3 |  |
| F4 | C | 3 | 3 |
| F5 | A | 3 | 10 |
|  | B | 3 |  |
|  | C | 4 |  |
| F6 | A | 3 | 8 |
|  | B | 3 |  |
|  | C | 2 |  |
| F7 | A | 4 | 16 |
|  | B | 4 |  |
|  | C | 4 |  |
|  | C | 4 |  |
| F8 | A | 3 | 7 |
|  | B | 2 |  |
|  | C | 2 |  |
| F10 | B | 3 | 6 |
|  | C | 3 |  |
| F11 | A | 5 | 10 |
|  | B | 5 |  |
| F12 | A | 4 | 8 |
|  | B | 4 |  |
| F13 | A | 3 | 6 |
|  | B | 3 |  |
| F14 | A | 3 | 10 |
|  | B | 3 |  |
|  | C | 3 |  |
|  | D | 1 |  |
| F15 |  |  | 10 |
| F16 |  |  | 13 |
| F17 |  |  | 10 |
