# Supplementary material for: Assessment of pathogens in flood waters in coastal rural regions: Case study after Hurricane Michael and Florence
Source: PLoS One. 2023 Aug 4;18(8):e0273757. doi: 10.1371/journal.pone.0273757 (PMC10403080; doi:10.1371/journal.pone.0273757)
Supplement: S2 Table — (DOCX) [file pone.0273757.s002.docx]

**Assessment of pathogens in flood waters in coastal rural regions: Case study after Hurricane Michael and Florence**

Moiz Usmani^1^, Sital Uprety^2^, Nathan Bonham^1^, Yusuf Jamal^1^, Yuqing Mao^2^, Daisuke Sano^3^, Joanna Shisler^4,5^, Avinash Unnikrishnan^6^, Thanh H. Nguyen^2^, Antarpreet Jutla^1^*

^1^Environmental Engineering Sciences, University of Florida, Gainesville, FL, USA

^2^Department of Civil and Environmental Engineering, University of Illinois at Urbana-Champaign, Urbana, IL, USA

^3^Department of Civil and Environmental Engineering, Tohoku University, Sendai, Japan

^4^Institute for Genomic Biology, University of Illinois at Urbana-Champaign, Urbana, IL, USA

^5^Department of Microbiology, University of Illinois at Urbana-Champaign, Urbana, IL, USA

^6^Civil and Environmental Engineering, Portland State University, Portland, OR, USA

*Corresponding author: ajutla@ufl.edu

Supplementary Material Table 2

Table S2: List of genes and primers used for pathogens tested in the study.

| Target organism | Target gene | Primer name | Primer sequence (5'-->3") | Reference |
| --- | --- | --- | --- | --- |
| *Enterococcus* spp. | 23S rRNA | ECST748F | GAGAAATTCCAAACGAACTTG | ^2^ |
|  |  | ENC854R | CAGTGCTCTACCTCCATCATT |  |
|  |  | GPL813TQ | FAM-TGGTTCTCT/ZEN/CCGAAATAGCTTTAGGGCTA-IBFQ |  |
| General *E. coli* | *ftsZ* | ftsZ_973F | CTGGTGACCAATAAGCAGGTT | ^3^ |
|  |  | ftsZ_1032R | CATCCCATGCTGCTGGTAG |  |
|  |  | UPL71 | Roche |  |
|  | *uidA* | uidA_993F | CCCTTACGCTGAAGAGATGC | ^3^ |
|  |  | uidA_1053R | TTCATCAATCACCACGATGC |  |
|  |  | UPL113 | Roche |  |
| Enteropathogenic *E. coli* (EPEC) | *eaeA* | eaeA_877F | GGCGAATACTGGCGAGACTA | ^3^ |
|  |  | eaeA_976R | GGCGCTCATCATAGTCTTTCTT |  |
|  |  | UPL28 | Roche |  |
| Shiga-toxin producing *E. coli* (STEC) | *stx*_1_ | stx1_636F | GCGTGGGTATTAATGAGTTGG | ^3^ |
|  |  | stx1_711R | TCATCTCGTTCAGTACGGTGTATT |  |
|  |  | UPL60 | Roche |  |
|  | *stx*_2_ | stx2_483F | TGTAATGACTGCTGAAGATGTTGAT | ^3^ |
|  |  | stx2_560R | TCCATGATARTCAGGCAGGA |  |
|  |  | UPL126 | Roche |  |
| *Shigella* spp. | *ipaH* 7.8 | ipaH_81FF | TCTGAGAATCCTGACTGAATGG | ^3^ |
|  |  | ipaH_142R | AAGCAATGCCTCGCTCTTC |  |
|  |  | UPL7 |  |  |
|  | *ipaH* all | ipaH_1136F | AAGGCCTTTTCGATAATGATACC | ^3^ |
|  |  | ipaH_1202R | ATTTCGAGGCGGAACATTT |  |
|  |  | UPL108 | Roche |  |
| *Shigella flexneri* | *virA* | virA_836F | GGCAATCTCTTCACATCACG | ^3^ |
|  |  | virA_897R | TTCGGACATAATTTGGGCATA |  |
|  |  | UPL6 | Roche |  |
| *Campylobacter jejuni* | *cadF* | cadF_267F | TGCTATTAAAGGTATTGATGTRGGTGA | ^3^ |
|  |  | cadF_350R | GCAGCATTTGAAAAATCYTCAT |  |
|  |  | UPL39 | Roche |  |
|  | *ciaB* | ciaB_718F | GCGTTTTGTGAAAAAGATGAAGATAG | ^3^ |
|  |  | ciaB_797R | GGTGATTTTACTTTCATCCAAGC |  |
|  |  | UPL137 | Roche |  |
| *Campylobacter lari* | *bipA* | Campy2f(Cla) | CATTTCAGCTTTTCTTTTGCCTAGT | ^4^ |
|  |  | Campy2r(Cla) | AAAACCGAACCATTTGAACACTTAG |  |
|  |  | CAMPY2pr | FAM-ACCACACCA/ZEN/GTAAAATCATCAGGCACATCA-IBFQ |  |
| *Salmonella* Typhimurium | *invA* | invA_176F | CAACGTTTCCTGCGGTACTGT | ^3^ |
|  |  | invA_291R | CCCGAACGTGGCGATAATT |  |
|  |  | invA_FAM_208 | FAM-CTCTTTCGT/ZEN/CTGGCATTAT-IBFQ |  |
|  | *ttrC* | ttrC_440F | ATTTTTGGCAGCCTTACCG | ^3^ |
|  |  | ttrC_507R | GCCTTACAGGCGTTCTTCG |  |
|  |  | UPL149 | Roche |  |
| *Clostridium perfringens* | *cpe* | cpe_823F | GAACAGTCCTTAGGTGATGGAGTAA | ^3^ |
|  |  | cpe_914R | GATGAATTAGCTTTCATTACAAGAACA |  |
|  |  | UPL159 | Roche |  |
| *Legionella pneumophila* | *mip* | mip_99F | GGATAAGTTGTCTTATAGCATTGGTG |  |
|  |  | mip_172R | CCGGATTAACATCTATGCCTTG | ^3^ |
|  |  | UPL60 | Roche |  |
| *Listeria* *monocytogenes* | *iap* | iap_1359F | TGGCGTTAAATACGATAACATCC | ^3^ |
|  |  | iap_1421R | CGACCGAAGCCAACTAGATATT |  |
|  |  | UPL106 | Roche |  |
|  | *hlyA* | Lm_hlyA_232F | TACCACGGAGATGCAGTGAC | ^3^ |
|  |  | Lm_hlyA308R | TTCTCCACAACAATATATTCATTTCC |  |
|  |  | UPL142 | Roche |  |
| *Vibrio cholerae* | *ctxA* | VC_ctxAF | TTTGTTAGGCACGATGATGGAT | ^5^ |
|  |  | VC_ctxAR | ACCAGACAATATAGTTTGACCCACTAAG |  |
|  |  | VC_ctxA_MGB | FAM-TGTTTCCAC/ZEN/CTCAATTAGTTTGAGAAGTGCCC-IBFQ |  |
|  | *toxR* | VC_toxR_420/334F | GTTTGGCGWGAGCAAGGTTT | ^3^ |
|  |  | VC_toxR_585R | TCTCTTCTTCAACCGTTTCCA |  |
|  |  | toxR_464/378FAM | FAM-CGCAGAGTM/ZEN/GAAATGGCTTGG-IBFQ |  |
| *Vibrio paraheamolyticus* | *tdhS* | VP_tdhF | AAACATCTGCTTTTGAGCTTCCA | ^6^ |
|  |  | VP_tdhR | CTCGAACAACAAACAATATCTCATCAG |  |
|  |  | VP_tdhS_MGB | FAM-TGTCCCTTT/ZEN/TCCTGCCCCCGG-IBFQ |  |
| *Pseudogulbenkiania* sp. NH8B | NH8B_3641 | IAC_23F | CAGGCCGTGAAGTCAAGC | ^3^ |
|  |  | IAC_92R | GAGGCGATGTGGATGGTC |  |
|  |  | UPL56 | Roche |  |
| *Giardia Lambia* | b-Giardin P241 | b-Giardin_241F | CATCCGCGAGGAGGTCAA | ^7^ |
|  |  | b-Giardin_241R | GCAGCCATGGTGTCGATCT |  |
|  |  | b-Giardin_241FAM | FAM/AAGTCCGCCGACAACATGTACCTAACGA/BHQ-1 |  |
